# Supplementary material for: Optimization of highly efficient exogenous-DNA-free Cas9-ribonucleoprotein mediated gene editing in disease susceptibility loci in wheat (Triticum aestivum L.)
Source: Front Plant Sci. 2023 Jan 10;13:1084700. doi: 10.3389/fpls.2022.1084700 (PMC9872142; doi:10.3389/fpls.2022.1084700)
Supplement: Supplementary Table 1 — gRNA target sequences. [file Table_1.pdf]

gRNA ID	Target Site Sequence
Pi21gD	ACAACAGGGTGATCGTCCGT
Tsn1g2	GGAACTGTCTACTAATATAT
Tsn1g3	ACCATAAAGGGGATTTGTGA
Snn5g1	ATACAATGGAGAACTAGTTA
Snn5g2	CTTGCAAGGACTTGATGATA

Table S1
