# Supplementary material for: Optimization of highly efficient exogenous-DNA-free Cas9-ribonucleoprotein mediated gene editing in disease susceptibility loci in wheat (Triticum aestivum L.)
Source: Front Plant Sci. 2023 Jan 10;13:1084700. doi: 10.3389/fpls.2022.1084700 (PMC9872142; doi:10.3389/fpls.2022.1084700)
Supplement: Supplementary Table 2 — Primers used to amplify the target region for amplicon next generation sequencing. Nucleotides shown in capital letters are the 5′-stub compatible with Illumina NGS library preparation. [file Table_2.pdf]

| **Amplicon target** | **F Primer** | **R Primer** |
|---|---|---|
| Pi21gD | GCTCTTCCGATCTagttcttcttacgtaagattgatcata | GCTCTTCCGATCTcaggccttgcaccagatctt |
| Tsn1g2 / Tsn1g3 | GCTCTTCCGATCTggaaactgatttctc | GCTCTTCCGATCTcaaaatccgccagtt |
| Snn5g1 | GCTCTTCCGATCTtgacagtgaattccgtaacc | GCTCTTCCGATCTtagtaatgtggagcaccttc |
| Snn5g2 | GCTCTTCCGATCTgctgactacaaacagattgtcc | GCTCTTCCGATCTtaactatttggtagcagtagcc |

**Table S2**
