# Supplementary material for: Optimization of highly efficient exogenous-DNA-free Cas9-ribonucleoprotein mediated gene editing in disease susceptibility loci in wheat (Triticum aestivum L.)
Source: Front Plant Sci. 2023 Jan 10;13:1084700. doi: 10.3389/fpls.2022.1084700 (PMC9872142; doi:10.3389/fpls.2022.1084700)
Supplement: Supplementary Table 3 — Genotypes of all edited M0 plants obtained. + indicates the number of base pairs inserted, - indicates the number of base pairs deleted. [file Table_3.pdf]

**Target: Pi21gD**						
	Genome A		Genome B		Genome D	
Plant Number	Allele 1	Allele 2	Allele 1	Allele 2	Allele 1	Allele 2
1	+1	-14	+1	WT	+1	WT
2	WT	WT	WT	WT	-5	WT
3	-1	-4	-1	-1	+1	+1
4	WT	WT	+1	WT	+1	WT
5	+1	+1	-6	-5	-1	+1

**Target: Tsn1g2**		
	Genome B	
Plant Number	Allele 1	Allele 2
1	-2	-5
2	-2	WT
3	-5	WT
4	-2	WT
5	-2	WT
6	-5	WT
7	-3	-15
8	-1	-1
9	-5	WT
10	-5	WT
11	-2	WT

**Target: Tsn1g3**		
	Genome B	
Plant Number	Allele 1	Allele 2
1	-31	WT
2	-1	WT
3	-1	WT
4	+1	WT
5	-2	-1
6	-1	-1
7	-2	WT
8	-1	WT
9	-2	-2

**Target: Snn5g1**		
	Genome B	
Plant Number	Allele 1	Allele 2
1	-5	WT
2	+20	WT
3	-11	-4
4	-8	-2
5	-10	-10
6	-42	-1
7	+1	-2
8	-4	WT
9	+2, -1	WT
10	-5	WT
11	-5	WT
12	-4	WT
13	-4	WT
14	-4	WT

**Target: Snn5g2**		
	Genome B	
Plant Number	Allele 1	Allele 2
1	-3	WT
2	-5	-1
3	-8	-3
4	-6	WT
5	-11	-6
6	-5	-1
7	-5	-1
8	-3	WT
9	-6	WT
10	-3	WT

Table S3
