# Supplementary figures and images for: Optimization of highly efficient exogenous-DNA-free Cas9-ribonucleoprotein mediated gene editing in disease susceptibility loci in wheat (Triticum aestivum L.)
Source: Front Plant Sci. 2023 Jan 10;13:1084700. doi: 10.3389/fpls.2022.1084700 (PMC9872142; doi:10.3389/fpls.2022.1084700)

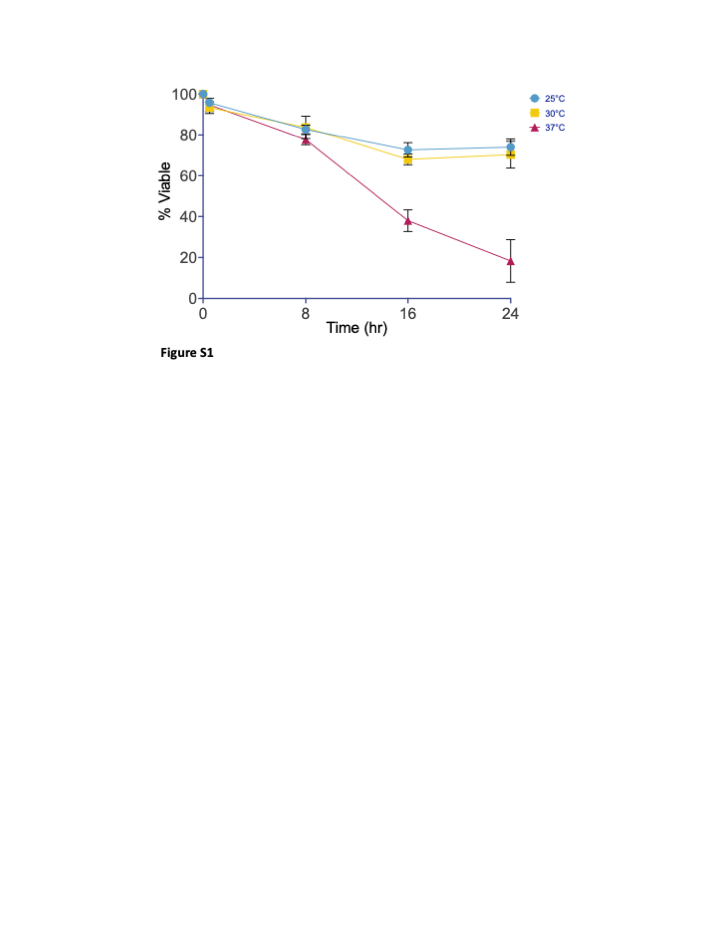

Supplement: Supplementary Figure 1 — Protoplast viability curve. N=3. Error bars indicate SEM. [file Image_1.png]

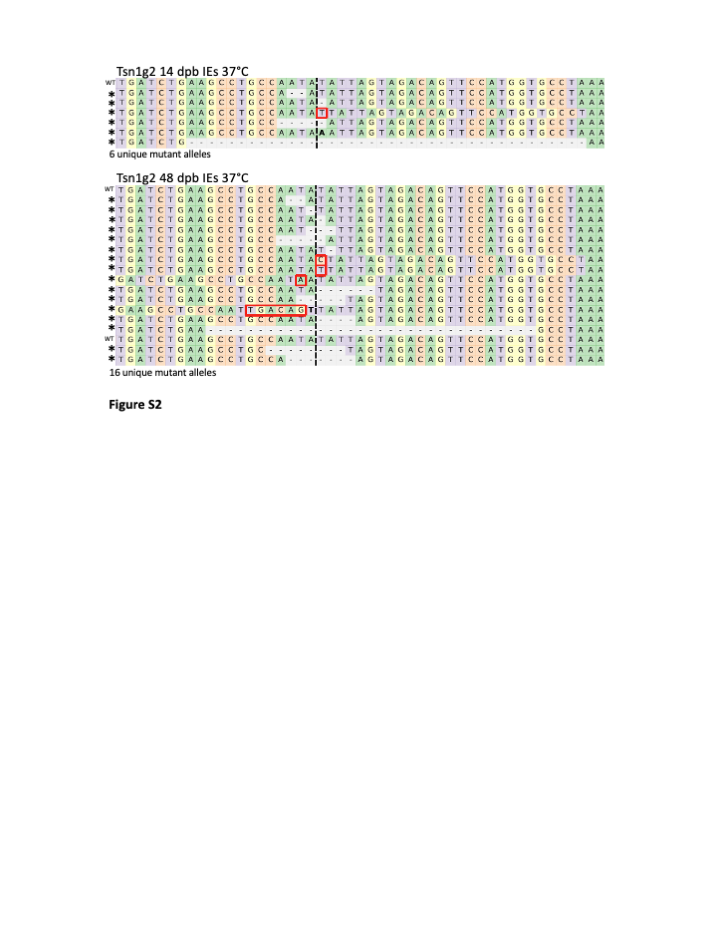

Supplement: Supplementary Figure 2 — Example of the difference in the number of unique mutant alleles between 14 dpb and 48 dpb. Provided are the detected alleles in immature embryos bombarded with Tsn1g2-Cas9 RNPs and treated at 37°C. The vertical bold dashed line represents the Cas9 cleavage site. Mutant alleles are marked with *. Wild type alleles are marked as WT. Dashes indicate base pair deletions, red boxes indicate base pair insertions, and bold letters indicate base pair substitutions. [file Image_2.png]

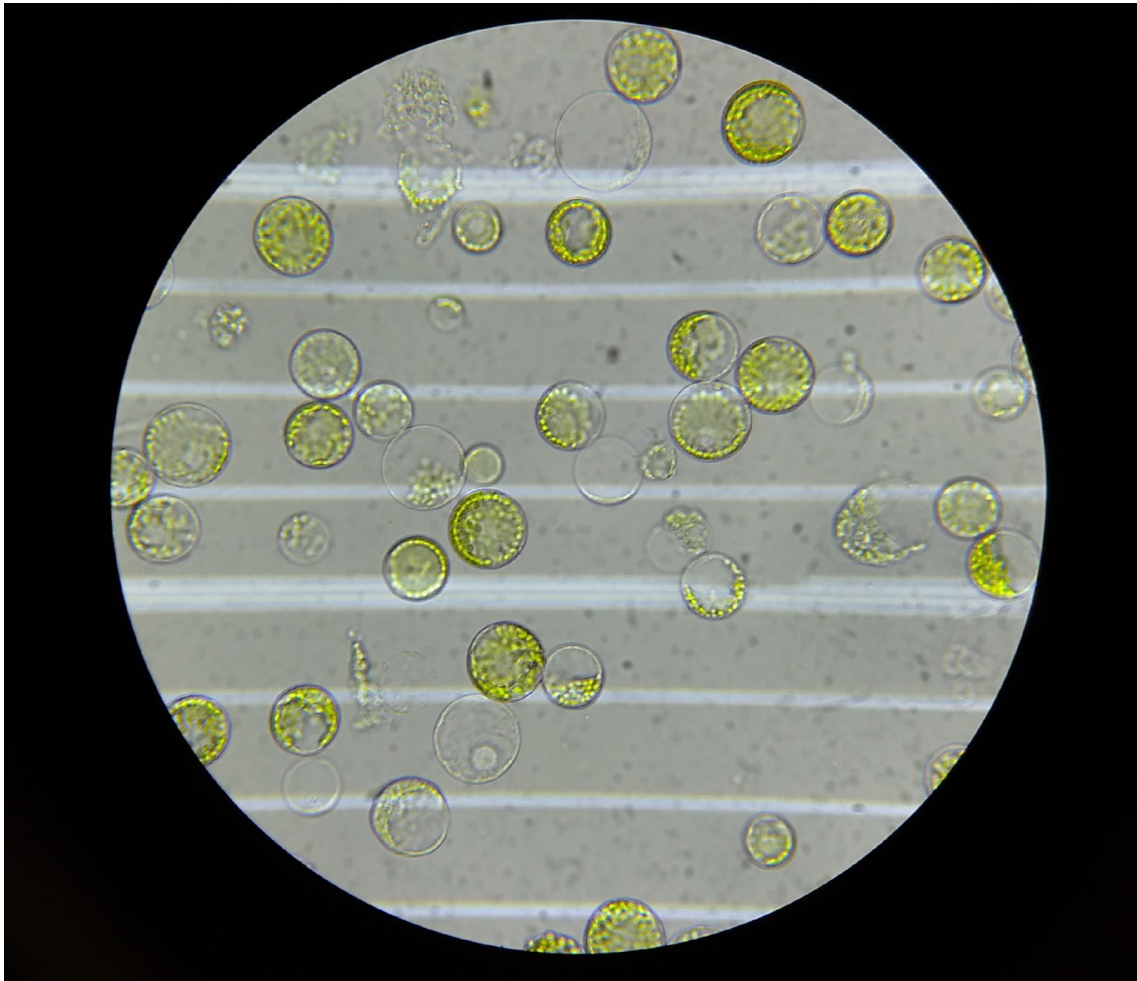

Figure S3

Supplement: Supplementary Figure 3 — Wheat protoplasts isolated from leaf tissue. [file Image_3.pdf]
